# Why Hysterectomy Algorithms Diverge from Clinical Practice: A Romanian Cohort Study

**DOI:** 10.3390/jcm15176617

**Published:** 2026-08-27

**Authors:** Nicoleta Alina Mareș, Alexandru Iordache, Niculae Iordache, Andrei Răzvan Stoica, Iulia Bistriceanu, Iuliana Ceausu, Cristian Viorel Poalelungi

**Affiliations:** 1 Faculty of Medicine, Carol Davila University of Medicine and Pharmacy, 37 Dionisie Lupu Street, 020021 Bucharest, Romania; nicoleta-alina.mares@drd.umfcd.ro (N.A.M.); a_iordache@yahoo.com (A.I.); cristian.poalelungi@umfcd.ro (C.V.P.); 2 Department of Obstetrics and Gynaecology, “Dr. I. Cantacuzino” Clinical Hospital, 5-7 Ion Movila, 030167 Bucharest, Romania; 3 General Surgery Department, “St. John” Clinical Emergency Hospital, 13 Vitan-Barzesti, 042122 Bucharest, Romania; 4 The Academy of Romanian Scientists, Ilfov 3 Street, 030167 Bucharest, Romania

**Keywords:** hysterectomy, surgical approach, postoperative recovery, Romania, minimally invasive surgery

## Abstract

**Background**: Hysterectomy is a prevalent gynecological procedure and ensuring appropriate use of minimally invasive approaches is essential to enhance the value and quality of care. The aim of this study was to investigate the gap between algorithm-guided recommendations and routine surgical practice in a Romanian real-world cohort by identifying non-clinical factors associated with deviation from the algorithm, and assessing its impact on postoperative hospitalization. **Methods**: This is a retrospective analytical observational study conducted over a 4-year period (2021–2025). The database comprised 961 cases of hysterectomy performed for benign gynecological conditions using laparoscopic, abdominal and vaginal approaches in two tertiary centers. **Results**: The previously developed decision algorithm was implemented in a selected subgroup of approximately 332 (34.54%) patients, while the remaining 629 (65.45%) hysterectomies were performed according to surgeon preference or institutional resource availability. The mean duration of hospitalization of the entire lot was 6.88 days (SD = 2.70). In laparoscopic surgery, deviation was not associated with prolonged hospitalization, with results indicating a null effect, OR 1.08 (95% CI 0.72–1.62). In contrast, in the abdominal cohort, deviation was strongly associated with prolonged LOS, with OR 2.76 (95% CI 1.75–4.40). **Conclusions**: Patient-related factors shape the theoretical suitability of a surgical approach, but operator- and institution-related factors determine whether that option is realistically available. Strengthening training in minimally invasive surgery across all clinics could reduce selection bias.

## 1. Introduction

Hysterectomy is a prevalent gynecological procedure and ensuring appropriate use of minimally invasive approaches is essential to enhance the value and quality of care. For benign indications, vaginal and laparoscopic approaches are generally associated with reduced morbidity compared with open abdominal hysterectomy. Although laparoscopic procedures may have a longer operative time, this approach is generally associated with faster recovery, reflected in reduced analgesic requirements and a shorter average hospital stay. Moreover, studies show that even in obese patients, laparoscopy may shorten operative time compared with the open approach [1,2].

Current recommendations favor minimally invasive approaches, prioritizing vaginal hysterectomy when feasible and considering laparoscopy as an appropriate alternative when the vaginal route is not suitable. Nevertheless, abdominal hysterectomy continues to play an important role in patients for whom minimally invasive techniques are contraindicated or cannot be performed safely [3,4].

In a previous study published in 2026, we proposed and evaluated a decision-making algorithm for selecting the surgical route of hysterectomy, without demonstrating a reduction in the overall rates of abdominal hysterectomy following the implementation of the treatment algorithm. The selection of surgical approach was itself influenced by non-patient-related factors [5].

Although international guidelines suggest criteria for selecting the optimal route of total hysterectomy [3,6], real-world practice often departs from these recommendations. Over the past decade, no study has systematically examined the determinants of this divergence or its implications for postoperative outcomes. The existing literature primarily documents variability in practice. To our knowledge, this is the first Romanian study to investigate why recommended hysterectomy algorithms diverge from real-world surgical practice by examining the factors that ultimately shape the choice of surgical route for total hysterectomy.

Recently, the literature has increasingly shifted toward implementation science, real-world evidence, barriers to adopting minimally invasive techniques, and institutional variability in surgical practice. The rates of hysterectomy vary significantly between centers, as highlighted in a 10-year study from Germany [7], and surgeon expertise is the major determinant of the surgical approach, not the clinical criteria, as suggested by Whiteside et al. in 2024 [8]. A recent survey of over 150 gynecologists, all ISGE members, found that, despite broad awareness of existing guidelines, limited residency training and insufficient surgical experience remain the most frequently perceived barriers to minimally invasive hysterectomy [9].

In a recent systematic analysis published in the Romanian journal *Chirurgia*, we underscored that the absence of a clear consensus on standardized selection criteria for hysterectomy in benign pathology generates substantial variability in real-world practice, with decisions that do not always reflect the specific characteristics of each case. In the absence of universally accepted criteria, surgeons rely on their own experience and on the resources available, which leads to marked differences between institutions. Additionally, the lack of minimally invasive resources limits the application of the guidelines [10].

Clinical decision algorithms may reduce unwarranted variation in the selection of the surgical route for total hysterectomy. However, the implementation of such algorithms may itself be selective and influenced by factors unrelated to the patient’s clinical characteristics. As shown in recent analyses, the choice of surgical approach is shaped by both the patient’s clinical profile and the surgical team’s experience and individual preferences [11]. Thus, the theoretically most appropriate route for a given patient may not always be the route ultimately performed in routine clinical practice. Accurate case documentation is fundamental to maintaining quality standards and ensuring equity in gynecological care [12].

The aim of this study was to investigate the gap between algorithm-guided recommendations and routine surgical practice in a Romanian real-world cohort by identifying non-clinical factors associated with deviation from the algorithm, and assessing its impact on postoperative hospitalization.

## 2. Materials and Methods

### 2.1. Ethical Consideration

The analysis of the data in this study was conducted in accordance with the Declaration of Helsinki and approved by the Ethics Committee of “St John” Clinical Emergency Hospital, Bucharest, Romania (protocol code R612/23 January 2026), and the Ethics Committee of “Dr. Ion Cantacuzino” Clinical Hospital, Bucharest, Romania (protocol code 13562/30 June 2025). The ethics approvals listed refer to authorization for retrospective data analysis, not for data collection.

### 2.2. Study Design

This is a retrospective analytical observational study carried out over a 4-year period (November 2021–June 2025) and comprises cases from two university centers: the Surgery Department of “St John” Clinical Emergency Hospital and the Gynecology Department of “Dr. Ion Cantacuzino” Clinical Hospital. Including cases from distinct specialties strengthens the study by providing an interdisciplinary perspective and enhancing the external relevance of the findings.

### 2.3. Data Sources and Participants

The database comprised 961 cases of hysterectomy performed for benign gynecological conditions using laparoscopic (LH), vaginal (VH), and abdominal (AH) approaches in two tertiary centers. This study used pre-existing anonymized data collected retrospectively in both centers and no individual informed consent was required. Written informed consent was obtained from all patients at the outset.

Of the total cases, 332 procedures followed the algorithm’s recommended route, whereas the remaining 629 hysterectomies were performed according to surgeon preference or institutional resource availability, introducing a potential selection bias. Overall, the cohort included 450 laparoscopic hysterectomies, 416 open surgeries, and 95 vaginal hysterectomies (Table 1). Concomitant adnexectomy, whether unilateral or bilateral, was not included in the analysis; only the surgical approach was evaluated.

### 2.4. Working Protocol

The study uses existing data, without intervention in patient management. All data were anonymized prior to analysis. Cases were identified through institutional surgical databases, and data extraction was carried out by two independent reviewers to ensure accuracy. Discrepancies were resolved through consensus. The present analysis includes the entire cohort of benign hysterectomies recorded in the institutional database over the 4-year interval, with no exclusion criteria applied.

The database captured whether the evidence-based hysterectomy route selection algorithm published in 2026 (10.3390/life16050749) was applied during surgical planning and documented the specific reasons for non-application. Therefore, the algorithm was not developed using a separate dataset, and the concept of distinct development versus evaluation cohorts does not apply. Patients were classified according to whether the previously developed hysterectomy route selection algorithm was implemented in the clinical decision-making process. Adherence to the algorithm was defined as complete agreement between the surgical approach recommended by the decision algorithm and the route actually performed. Deviation from the algorithm was defined as any situation in which the surgical approach was chosen differently.

Data extraction included demographic characteristics, clinical variables, and non-clinical factors (surgical approach, operator-related factors (surgeon preference, laparoscopic expertise), and institutional constraints).

### 2.5. Study Objectives

The primary objective was to characterize the non-clinical factors associated with deviation from the recommended decision algorithm and to quantify the proportion of cases in which the algorithm was not followed.

The secondary objective was to assess the impact of deviation from the algorithm on postoperative recovery by comparing mean length of stay across adherence groups. Mean length of stay was initially explored descriptively (mean = 6 days), and prolonged hospitalization (>6 days) was selected as the analytical endpoint based on the distribution of LOS in our cohort.

### 2.6. Statistical Analyses

Data processing and initial organization were carried out using Microsoft Excel and statistical analyses were performed using IBM SPSS version 23.0. Categorical variables were analyzed using the χ^2^ (chi-squared) test. Continuous variables were reported as mean ± standard deviation or median and interquartile range, depending on distribution. Statistical significance was defined as *p* < 0.05 for all tests. No missing data were present in the dataset.

## 3. Results

Among 961 consecutive hysterectomies, the previously developed decision algorithm was implemented in a selected subgroup of approximately 332 (34.54%) patients, while the remaining 629 (65.45%) hysterectomies were performed according to surgeon preference or institutional resource availability. Overall, the cohort included 450 (46.82%) laparoscopic hysterectomies, 416 (43.28%) open surgeries, and 95 (9.8%) vaginal hysterectomies (Table 1).

### 3.1. Frequency of Algorithm Deviation and Non-Clinical Determinants

The selective implementation of the algorithm created a clinically relevant difference between the population in which the algorithm was evaluated and the broader population undergoing hysterectomy in routine practice. Deviation from algorithm-guided decision-making seemed to arise from determinants unrelated to patient characteristics, including surgeon-related factors and the availability of institutional resources. This implementation gap may have contributed to the observed variation in the distribution of abdominal, laparoscopic, and vaginal hysterectomy.

To contextualize the magnitude of the implementation gap, we determine the proportion of cases that diverged from the decision pathway. Given the deviations from the proposed algorithm observed exclusively in the laparoscopic and abdominal approaches, the analysis was restricted to these two subgroups. Among the 416 abdominal hysterectomies, only 102 cases (24.51%) followed the algorithm, while the remaining 75.49% were performed according to surgeon-related factors or institutional constraints. The subset of laparoscopic hysterectomies that fully met all algorithm criteria for adherence includes 135 cases (30%). The remaining laparoscopic cases (n = 315) were performed under conditions not compatible with algorithm criteria. We acknowledge that restricting the adherent group to these 135 cases may introduce selection bias, as we mention in the Discussion section.

Analyzing the open-surgery subgroup based on the observations recorded in the initial database, we found that 59.87% cases deviated from the algorithm due to surgeon-related factors, whereas 38.85% were attributable to institutional constraints and 1.27% to other unspecified causes. The distribution of non-clinical factors contributing to deviation from the recommended decision pathway is presented in Table 2.

The initial database also included subjective notes documenting the reasons reported at the time of surgical planning. Among surgeon-related factors, in 120 cases (63.82%) the decision to perform open surgery was driven by the unavailability of a minimally invasive surgical team, while in 68 cases (36.17%) the deviation was explained by surgeon preference or limited experience in minimally invasive techniques. Regarding institutional factors, 65.57% of cases were associated with an overloaded operating schedule or emergencies that altered case sequencing, whereas 34.43% were linked to equipment unavailability or technical malfunction. These data indicate that deviation from the algorithm occurred in a substantial proportion of cases.

It should be highlighted that the deviation from the algorithm reflects the intrinsic challenges of standardizing a surgical approach. Surgeon-related expertise, patient-specific factors, and institutional constraints inevitably shape intraoperative decision-making. As a result, the observed variability cannot be attributed solely to “selection bias”; rather, it reflects structural limitations inherent to applying a rigid decision pathway in real-world surgical practice.

### 3.2. Impact on Postoperative Recovery

The second objective aimed to evaluate the impact of deviation from the algorithm on postoperative recovery by analyzing the mean length of hospital stay (LOS). Among patients who underwent the surgical approach recommended by the decision algorithm, the analysis of LOS (n = 332) revealed a remarkably uniform central tendency across the three techniques, with the median consistently at 6 days. The arithmetic means were likewise very similar, 6.55 days for the abdominal approach (SD = 1.85), 6.44 days for the laparoscopic approach (SD = 2.72), and 6.36 days for the vaginal approach (SD = 1.95), suggesting that the type of intervention did not substantially influence hospitalization duration. The 95% confidence intervals showed considerable overlap across groups, indicating no meaningful differences between the means.

Table 3 presents the descriptive analysis of LOS according to surgical approach: abdominal, laparoscopic, and vaginal.

The mean duration of hospitalization of the entire lot was 6.88 days (SD = 2.70). The association between the surgical approach (laparoscopic vs. abdominal) and prolonged LOS was analyzed using univariate logistic regression. The results are presented as odds ratios (ORs) with 95% confidence intervals. No additional variables were included in the model, considering the comparative design and the objective of evaluating the direct effect of the surgical approach on the outcome. Given the overall median LOS of 6 days, we analyzed the proportion of cases with hospitalization longer than 6 days in both the LH and AH groups. These data are presented in Table 4.

In the laparoscopic subgroup, among the 315 cases that did not follow the decision algorithm, 179 cases (56.82%) had a hospital stay longer than 6 days, while among algorithm-adherent cases, approximately half exceeded this threshold (54.81%). Statistical analysis showed that, in the laparoscopic group, deviation from the algorithm was not associated with prolonged hospitalization. The odds of extended LOS were similar between deviated and non-deviated cases (OR 1.08, 95% CI 0.72–1.62, *p* = 0.78).

In the subgroup of hysterectomies performed through an open approach, a hospital stay longer than 6 days was observed in nearly two-thirds of the 314 cases that did not follow the algorithm (64.96%), compared with only 41 cases (40.19%) among the 102 algorithm-adherent cases. In the abdominal cohort, deviation from the algorithm was significantly associated with prolonged hospitalization. Deviated cases had higher odds of extended LOS compared with non-deviated cases (OR 2.76, 95% CI 1.75–4.40, *p* < 0.0001). In conclusion, abdominal hysterectomy cases that deviated from the algorithm had nearly twice the odds of prolonged LOS compared with adherent cases. The confidence interval does not cross 1, indicating a robust effect, and the association is strongly statistically significant (*p* < 0.0001).

**Figure 1:**
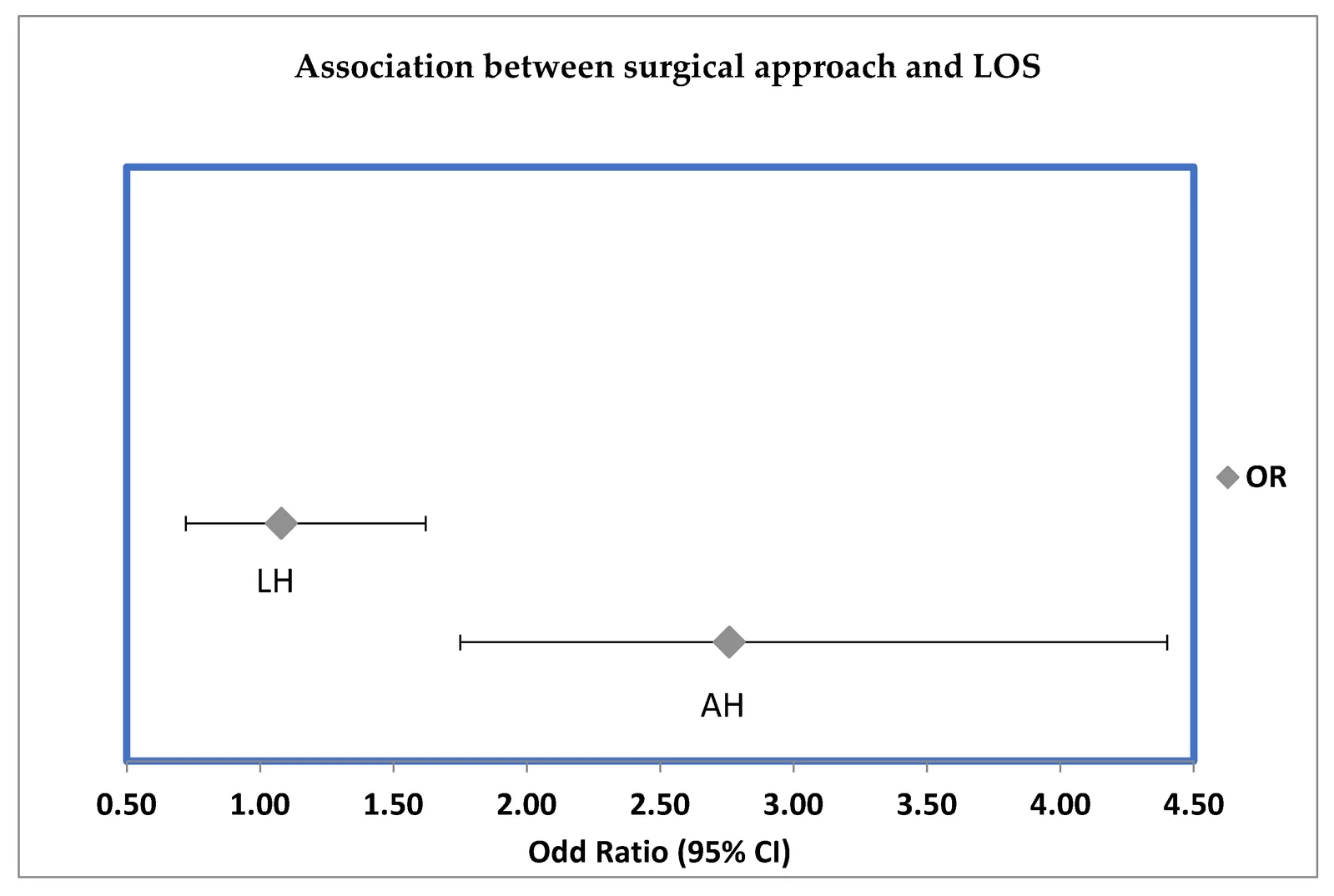
Forest plot showing the association between surgical approach and prolonged hospital stay. The horizontal axis represents the odds ratio (log scale) and 95% confidence intervals for laparoscopic hysterectomy (LH) and abdominal hysterectomy (AH).

The impact of algorithm deviation on postoperative recovery differed substantially between surgical approaches (Figure 1). In laparoscopic surgery, deviation was not associated with prolonged hospitalization, with results indicating a null effect, OR 1.08 (95% CI 0.72–1.62). This pattern suggests that, in a minimally invasive context, decision variability does not translate into measurable clinical consequences, likely due to accelerated recovery associated with the technique. In contrast, in the abdominal cohort, deviation was strongly associated with prolonged LOS, with OR 2.76 (95% CI 1.75–4.40), thereby raising the risk of delayed recovery.

Overall, the results support the utility of the selection algorithm and underscore the relevance of decision stratification, particularly for abdominal cases. In summary, deviation from the recommended route was significantly associated with prolonged hospitalization, suggesting a measurable clinical impact. We found that divergence from the recommended route occurred in nearly two-thirds of cases, predominantly driven by limited surgeon expertise and institutional resource constraints, with significant consequences for postoperative recovery. This bias limits both the generalizability of the algorithms and the comparability of postoperative outcomes between groups.

## 4. Discussion

The literature can be broadly classified into four categories: work documenting variation in practice across regions, hospitals, or surgeons; analyses proposing or evaluating decision algorithms; investigations of perceived barriers such as limited training or insufficient familiarity with minimally invasive techniques; and research demonstrating the influence of surgeon preference on decision-making.

Published evidence indicates that open surgical approaches for hysterectomy remain overused even in cases where minimally invasive techniques could be appropriately employed. Limiting unnecessary open hysterectomies yields clear benefits, including faster recovery, lower rates of complications, and reduced healthcare costs [13,14,15,16]. Although decision algorithms have been proposed for selecting the approach method in hysterectomy, their application in clinical practice is often limited by external factors such as the availability of technological resources, the surgeon’s level of experience, or the institution’s preferences. This large, dual-center Romanian cohort shows that algorithm-recommended hysterectomy routes diverge from actual surgical practice in nearly two-thirds of cases. These findings show a substantial disconnect between guideline-based recommendations and real-world practice, underscoring the influence of structural determinants that extend beyond patient-level clinical criteria.

One way of reducing variability in care is by adopting clinical pathways. Kovacs reported in 2002 that applying route selection guidelines shifted hysterectomy practice toward more vaginal procedures and fewer abdominal ones, reducing variability in clinical practice [17]. A 2017 study of 6569 benign hysterectomies evaluated the impact of implementing a standardized pathway and found reductions in adverse outcomes and complications. However, it did not assess case-level discordance between the recommended and actual surgical route, nor the reasons for which the algorithm was not applied [18].

Although the standardization of decision-making is often proposed as a means to improve consistency in clinical practice, the specific factors influencing route selection remain insufficiently clarified. A 2019 analysis of 230,876 hysterectomies demonstrated substantial temporal and regional variation in the use of abdominal, vaginal, and laparoscopic approaches, underscoring that variability in surgical route selection reflects a broader system-level phenomenon rather than a pattern confined to individual institutions [19]. A recent 2024 study from the Netherlands similarly revealed marked regional variation in hysterectomy rates and surgical route selection, indicating that a patient’s likelihood of receiving a particular approach can differ substantially depending on the regional context and institutional environment [20].

Early studies suggested that physician preference may influence the selection of the surgical route beyond differences in patient characteristics. A 1995 retrospective analysis of 502 patients treated by 16 gynecologists showed that abdominal hysterectomies were sometimes performed without clear clinical indication, indicating that variability in route selection cannot be explained solely by patient characteristics and that surgeon practice style and personal preferences play a meaningful role [21]. Similarly, a population-based administrative study from 1996 showed that physician-related factors contributed significantly to variation in hysterectomy practice, adding explanatory power beyond patient and hospital characteristics and underscoring the meaningful role of non-clinical determinants [22].

In a previous study, we explicitly discussed practitioner variability and the fact that approach selection is influenced by technology and context; however, the objective was to develop the algorithm rather than systematically measure the divergence between recommended approaches and actual practice. The data analysis identified determinants of surgical approach selection, and tried to validate the proposed decision algorithm in a real-world, heterogeneous population [5]. A 2017 study examined the implementation of a pathway aimed at reducing abdominal hysterectomies and explored both surgeon-related and system-related factors [23]. Conversely, a practice survey assessed the approaches surgeons use, their preferences, and the factors they consider, yet did not provide an applied algorithm or a measurement of deviation from it [9].

A German study from 2020 showed that hysterectomy route selection has shifted markedly over the past decade, driven not only by patient characteristics but also by contextual and institutional factors such as infrastructure, team expertise, and local practice trends, resulting in substantial variation across centers [7]. Also in 2020, Schmitt et al. evaluated a hysterectomy selection algorithm integrating key clinical factors and found that, among 365 patients, 12.6% of procedures diverged toward a more invasive route than recommended, suggesting that prospective nationwide use of such tools may help reduce unwarranted variation and associated healthcare costs [24].

Many studies have identified insufficient residency training and limited surgical experience as the predominant barriers to performing minimally invasive hysterectomy, strongly supporting the concept of operator-related constraints in real-world decision-making [25,26]. Similarly, Janda et al. (2018), in a survey of 258 specialists, demonstrated that the lack of structured training opportunities and insufficient laparoscopic skill acquisition continued to represent the main impediments to expanding the use of laparoscopic hysterectomy [27]. Together, these findings highlight a persistent gap between evidence-based recommendations and actual surgical practice, driven largely by variability in surgeon expertise and training exposure, as we have concluded.

Whiteside et al. similarly showed that surgeon expertise and access to appropriate technology are major determinants of hysterectomy route selection, at times outweighing the clinical indication itself [8]. These observations align closely with our findings, in which limited competence in laparoscopic or vaginal techniques and the unavailability of necessary equipment represented the principal reasons for deviation from the algorithm-recommended route. Surgeon experience in laparoscopic hysterectomy is pivotal for minimizing complications, making rigorous and structured training indispensable to ensure competent and safe performance, as all laparoscopic procedures require achieving an optimal learning curve [28,29].

Other studies have emphasized that insufficient training remains a major barrier, as the higher technical demands of laparoscopic hysterectomy restrict its routine use among many gynecologists. Consequently, developing competence in minimally invasive surgery should become a priority within advanced surgical training modules. Nonetheless, limited access to this technique is not determined solely by surgeon skill, as broader structural factors also influence the feasibility of offering laparoscopic hysterectomy [30,31]. The substantial overlap between laparoscopic and abdominal approaches across many clinical profiles underscores the influence of local surgical capacity and operator expertise on final route selection [5].

Expanding the use of minimally invasive procedures requires both the development of appropriate clinical pathways and adequate surgical training. The UK’s GIRFT (“Get It Right First Time”) program, which promotes surgery by the right surgeon, in the right place, at the right time, is designed not to limit clinical autonomy but to reduce variation in access to care and improve patient outcomes [32,33]. Tailored surgical management is essential, reflecting the core principle of personalized medicine [34].

Frequently reported barriers to minimally invasive hysterectomy include insufficient residency training, technical complexity, limited operative experience, longer operative times, and caseload constraints, underscoring that the surgical route chosen is shaped as much by institutional and surgical context as by patient-specific clinical factors [35]. Overall, algorithms improve candidate selection, but their effectiveness is resource-dependent [5,35]. This observation directly aligns with our findings.

Real-world biases in surgical practice are not isolated operator-level deviations but structural patterns shaped by resource limitations, variability in surgeon competence, and institutional preferences. Within this broader context, our study provides a pragmatic contribution by illustrating, through data from two Romanian hospitals, the practical limitations that hinder the effective implementation of such decision-making algorithms in routine clinical care. Surgical route selection is a multilevel decision occurring at the intersection of patient characteristics, provider capabilities, and institutional resources; that is why algorithms diverge from clinical practice. Importantly, such discordance should not be interpreted as inappropriate care. Clinical conditions may evolve, additional intraoperative findings may emerge, and both patient and clinician preferences may legitimately modify the initial plan. Ultimately, the surgeon’s judgment transforms guidelines into individualized patient care.

In summary, our findings show that decision algorithms alone cannot standardize hysterectomy practice when the expertise, infrastructure, and institutional conditions required for their implementation remain unevenly available. These results highlight the need for implementation strategies that address variability in training, resource allocation, and institutional support, underscoring the broader challenge of translating algorithm-guided recommendations into consistent clinical practice.

This study is limited by its single-country, two-center design, the retrospective nature of data collection, and the potential misclassification inherent in auditing resources and surgical expertise.

## 5. Conclusions

Patient-related factors determine the theoretical suitability of a surgical approach, but operator- and institution-related factors shape whether that option is realistically available in practice.

This first Romanian evaluation demonstrates that deviation from algorithm-guided recommendations was associated with system-level constraints, highlighting the gap between structured decision pathways and real-world surgical practice. Given the prolonged hospitalization observed in the abdominal subgroup, the open approach should be considered only for selected cases. Efforts to improve adherence should prioritize focused training in minimally invasive techniques, investment in equipment, and institutional policies that support guideline-concordant care.

## Figures and Tables

**Table 1:** Distribution of surgical approaches.

Surgical Approach	n	%
Abdominal (AH)	416	43.28
Laparoscopic (LH)	450	46.82
Vaginal (VH)	95	9.8
Total	961	100%

**Table 2:** Distribution of non-clinical determinants of algorithm deviation in AH subgroup.

Non-Clinical Factors	n	%
Surgeon	188	59.87
Resources	122	38.85
Others	4	1.27
Total	314	100%

**Table 3:** Descriptive analysis of the average length of hospital stay, depending on the type of surgical approach.

	LOS			
Surgical Approach	AH	LH	VH	Total (LOS)
Valid	102	135	95	332
Missing values	0	0	0	0
Median	6	6	6	6
Mean	6.56	6.45	6.37	6.46
Upper limit of the 95% CI for the mean	6.92	6.91	6.77	6.70
Lower limit of the 95% CI for the mean	6.19	5.98	5.97	6.21
Standard deviation (SD)	1.86	2.72	1.96	2.27
Upper limit of the 95% CI for the standard deviation	2.16	3.09	2.28	2.45
Lower limit of the 95% CI for the standard deviation	1.63	2.43	1.71	2.11

**Table 4:** Odds ratios (ORs) and 95% confidence intervals (CIs) for each surgical subgroup. “Error Left” and “Error Right” represent the lower and upper standard errors associated with each estimate.

Subgroup	OR	95% CI Low	95% CI High	Error Left	Error Right
LH	1.08	0.72	1.63	0.36	0.54
AH	2.76	1.75	4.40	1.01	1.64

## Data Availability

The original contributions presented in the study are included in the article; further inquiries can be directed to the corresponding author.

## Abbreviations

The following abbreviations are used in this manuscript:

ACOG	American College of Obstetricians and Gynecologists
LH	Laparoscopic Hysterectomy
AH	Abdominal Hysterectomy
VH	Vaginal Hysterectomy
LOS	Length of Hospital Stay
